# Identification of Haplotypes Associated With Resistance to Bacterial Cold Water Disease in Rainbow Trout Using Whole-Genome Resequencing

**DOI:** 10.3389/fgene.2022.936806

**Published:** 2022-06-23

**Authors:** Sixin Liu, Kyle E. Martin, Guangtu Gao, Roseanna Long, Jason P. Evenhuis, Timothy D. Leeds, Gregory D. Wiens, Yniv Palti

**Affiliations:** ^1^ National Center for Cool and Cold Water Aquaculture, Agricultural Research Service, United States Department of Agriculture, Kearneysville, WV, United States; ^2^ Troutlodge Inc., Sumner, WA, United States

**Keywords:** rainbow trout, bacterial cold water disease, haplotype, SNP, MAS, QTL, whole genome sequencing (WGS)

## Abstract

Bacterial cold water disease (BCWD) is an important disease in rainbow trout aquaculture. Previously, we have identified and validated two major QTL (quantitative trait loci) for BCWD resistance, located on chromosomes Omy08 and Omy25, in the odd-year Troutlodge May spawning population. We also demonstrated that marker-assisted selection (MAS) for BCWD resistance using the favorable haplotypes associated with the two major QTL is feasible. However, each favorable haplotype spans a large genomic region of 1.3–1.6 Mb. Recombination events within the haplotype regions will result in new haplotypes associated with BCWD resistance, which will reduce the accuracy of MAS for BCWD resistance over time. The objectives of this study were 1) to identify additional SNPs (single nucleotide polymorphisms) associated with BCWD resistance using whole-genome sequencing (WGS); 2) to validate the SNPs associated with BCWD resistance using family-based association mapping; 3) to refine the haplotypes associated with BCWD resistance; and 4) to evaluate MAS for BCWD resistance using the refined QTL haplotypes. Four consecutive generations of the Troutlodge May spawning population were evaluated for BCWD resistance. Parents and offspring were sequenced as individuals and in pools based on their BCWD phenotypes. Over 12 million SNPs were identified by mapping the sequences from the individuals and pools to the reference genome. SNPs with significantly different allele frequencies between the two BCWD phenotype groups were selected to develop SNP assays for family-based association mapping in three consecutive generations of the Troutlodge May spawning population. Among the 78 SNPs derived from WGS, 77 SNPs were associated with BCWD resistance in at least one of the three consecutive generations. The additional SNPs associated with BCWD resistance allowed us to reduce the physical sizes of haplotypes associated with BCWD resistance to less than 0.5 Mb. We also demonstrated that the refined QTL haplotypes can be used for MAS in the Troutlodge May spawning population. Therefore, the SNPs and haplotypes reported in this study provide additional resources for improvement of BCWD resistance in rainbow trout.

## Introduction

The global demand for seafood has roughly doubled since the start of the 21^st^ century, and will likely double again by 2050 (Naylor et al., 2021). Rainbow trout (*Oncorhynchus mykiss*) is one of the most widely cultured cold freshwater fish, with production on every continent except Antarctica. The global production of rainbow trout was about 917,000 tons in 2019 (FAO, 2021). Outbreaks of infectious disease are one of the major challenges for rainbow trout production and welfare. Bacterial cold water disease (BCWD), caused by *Flavobacterium psychrophilum*, is a frequent disease in rainbow trout (Nematollahi et al., 2003; Starliper, 2011; Loch and Faisal, 2015). Commercial vaccines for BCWD are not available yet. Use of licensed antibiotics for BCWD treatment increases production cost and antibiotic resistant pathogens may emerge.

Use of genetic resistance is an effective approach to control BCWD in rainbow trout. A rainbow trout line with improved resistance to BCWD has been developed by using family-based selection (Leeds et al., 2010; Wiens et al., 2013a; Wiens et al., 2018). Recently, multiple studies have demonstrated that genomic selection (GS) can substantially improve the accuracy of selection for BCWD resistance in rainbow trout. Vallejo et al. (2017a) reported that GS using a 57K SNP (single nucleotide polymorphism) genotyping array (Palti et al., 2015a) can double the accuracy of selection for BCWD resistance in a commercial breeding population. To reduce the cost of genotyping, the accuracy of GS for BCWD resistance was evaluated with low-density SNP panels. The accuracy of GS remained substantially higher than pedigree-based selection when using 70 SNPs associated with QTLs (quantitative trait locus) for BCWD resistance (Vallejo et al., 2018). To reduce the cost of BCWD phenotyping, it has recently been reported that the accuracy of GS for BCWD resistance without model retraining in the subsequent generation remained higher than pedigree-based selection (Vallejo et al., 2021).

To fully exploit the genetic resistance to BCWD, extensive genetic mapping studies were conducted to identify and validate QTLs for BCWD resistance in rainbow trout. Fraslin et al. (2018) used both immersion and intramuscular injection methods to evaluate double haploids derived from a cross between two rainbow trout isogenic lines, and 15 QTLs for BCWD resistance were identified. Also, two QTLs for BCWD resistance were identified after a natural disease outbreak on a French farm (Fraslin et al., 2019). At the USDA National Center for Cool and Cold Water Aquaculture, we initially used full-sib mapping families to identify and validate QTL for BCWD resistance (Wiens et al., 2013b; Vallejo et al., 2014a; Vallejo et al., 2014b; Palti et al., 2015b; Liu et al., 2015). With the advancement of genomic resources available in rainbow trout such as a SNP genotyping array (Palti et al., 2015a) and a reference genome (Pearse et al., 2019), we performed a genome-wide association study (GWAS) to detect QTL for BCWD resistance (Vallejo et al., 2017b) in the 2013 generation of the Troutlodge May spawning population. Three QTL for BCWD resistance with moderate-large effects, located on chromosomes Omy03, Omy08, and Omy25, were identified. In a follow-up study the three QTLs were validated in the 2015 generation of the Troutlodge May spawning population (Liu et al., 2018). In the same study it was shown that SNP haplotypes associated with the two major QTL on chromosomes Omy08 and Omy25 can be used for marker-assisted selection (MAS) for BCWD resistance. However, the two favorable haplotypes for the two major QTL on chromosomes Omy08 and Omy25 span regions of 1.3 and 1.6 Mb, respectively. Recombination events within the haplotype regions may result in new haplotypes associated with BCWD resistance, which will reduce the accuracy of MAS for BCWD resistance over time. Thus, it is important to identify and validate additional SNPs associated with the two major QTL for BCWD resistance with a goal to reduce the physical size of haplotypes associated with BCWD resistance.

Whole-genome sequencing (WGS) is a powerful tool to discover SNPs and to identify causative genes for traits of interest. With the recent rapid reduction in the cost of next generation sequencing, the use of WGS has become more common in genetic studies of salmonids (Gao et al., 2018; Narum et al., 2018; Thompson et al., 2020; Liu et al., 2021). The objectives of this study were 1) to identify additional SNPs associated with BCWD resistance using WGS; 2) to validate the SNPs associated with BCWD resistance using family-based association mapping; 3) to refine the haplotypes associated with BCWD resistance; and 4) to evaluate MAS for BCWD resistance using the refined QTL haplotypes.

## Materials and Methods

### Five Consecutive Generations of the Troutlodge May Spawning Strain

Troutlodge, Inc., has four rainbow trout strains (Liu et al., 2017) named by their peak spawning months. All samples used in this study were from the May spawning strain. Five consecutive generations (Table 1) were used in this study. The samples used for WGS were from the 2013 and 2015 generations. Selected families from three consecutive generations, 2015, 2017, and 2019, were used for association analyses of BCWD resistance. Fish of the 2021 generation were used to evaluate MAS for BCWD resistance. Previously, each Troutlodge strain had two populations, odd-year and even-year populations. The odd-year and even-year May spawning populations have been merged into one population since the 2019 generation.

**TABLE 1:** Summary of samples from five consecutive generations of the Troutlodge May spawning population used in this study.

Generation	Study	Comment
2013	WGS	Selected parents for the 2015 generation were used for WGS (Individuals and pools; N = 20)
2015	WGS, SNP validation	1) Selected parents (Individuals and pools; N = 20) and families (pools; N = 480) were used for WGS; 2) Validate SNPs associated with BCWD resistance using 60 families (10 offspring per family; N = 600) with intermediate BCWD survival rates
2017	SNP validation	Validate SNPs associated with BCWD resistance using 60 families (10 offspring per family; N = 600) with intermediate BCWD survival rates
2019	SNP validation	Validate SNPs associated with BCWD resistance using 9 families (92 offspring per family; N = 828) with intermediate BCWD survival rates
2021	MAS	Evaluation of MAS using Pooled BCWD challenge (50 families; 20 fish per family; N = 1,000)

### BCWD Challenge Experiments

BCWD challenge experiments were conducted in four consecutive generations, 2015, 2017, 2019, and 2021 (Supplementary Table S1). Fish (80–99 days post-hatch) were challenged by intraperitoneal injection of *Flavobacterium psychrophilum* strain CSF259-93 using the established protocol described in detail by Hadidi et al. (2008). Mortalities were collected daily for 21 days after intraperitoneal injection. Both survival days (DAYS), the number of days to death after BCWD challenge, and survival status (STATUS), 2 for dead fish and 1 for survivors at day 21, were recorded for each fish. Each family of the 2015 and 2017 generations was evaluated for BCWD resistance using two replicate tanks (3 L tank with a water flow rate of 1 L per minute) with 40 fish per tank, and the details have been reported in our previous publications (Vallejo et al., 2017a; Liu et al., 2018). For the 2019 generation, we increased the number of fish challenged per family to 3 or 4 replicate tanks (40 fish per tank) based on fish availability. The 2021 families were pooled and challenged as described below to evaluate MAS for BCWD resistance.

### Sequencing of 40 Parents of the 2015 and 2017 Generations

Based on the BCWD survival rates of the 2015 and 2017 families and parental haplotypes for the two targeted QTL regions, Omy08 and Omy25, we selected 40 (Supplementary Table S2) parents for individual sequencing and pooled sequencing. First, we sorted the families within each generation by BCWD survival rate from high to low. The parents of top 20 families were assigned to a BCWD resistant (R) group, and the parents of bottom 20 families were assigned to a BCWD susceptible (S) group. Then, the QTL haplotypes of these parents were reconstructed for the two QTL regions using the same SNP sets and method reported in Liu et al. (2018). The parents of the 2015 and 2017 generations were selected to target for the Omy08 and Omy25 BCWD QTL, respectively. For each generation, we selected 10 R parents that are fixed for the favorable haplotype for the targeted QTL and have at least one favorable haplotype for the other QTL. We also selected 10 S parents without any favorable haplotype for the targeted QTL. Thus, a total of 40 parents were selected for WGS with a targeted genome coverage of 15x per sample. In addition to sequencing of individuals, we also pooled equal amount of DNA per fish by BCWD groups within each generation for pooled sequencing. The targeted genome coverage per pool was 30x. The raw sequences of the parents were deposited in sequence read archive (SRA) under BioProject PRJNA681179.

### Sequencing of Pooled Offspring of the 2015 Generation

The sequencing of parents described above might be biased because both BCWD survival rates and QTL haplotypes were used to select the samples used for sequencing. Thus, we decided to use BCWD phenotype as the only criteria to select samples for additional pooled sequencing. Among the 138 families of the 2015 generation evaluated for BCWD resistance, we selected 60 families with intermediate BCWD survival rates that ranged from 24% to 71% for sequencing. For each of the 60 families, we selected the first four fish that died after day 3 (to avoid fish died from injection injury or stress) and four random survivors. Each of the four dead fish or survivors was randomly assigned to one of the four corresponding BCWD status pools. In total, we had four DNA pools of dead fish (S pools) and four DNA pools of survivors (R pools). Equal amount of DNA per sample was pooled for sequencing with a targeted genome coverage of 45x per DNA pool. The sequences of the pooled offspring were deposited under BioProject PRJNA830380.

### Whole-Genome Sequencing and SNP Identification

DNA was extracted from fin clips following the manufacturer’s recommended protocols for AutoGenprep 965 (Autogen, Holliston, MA, United States). Whole-genome DNA sequencing libraries were prepared using the KAPA HyperPrep kit (KAPA Biosystems, Wilmington MA), and were sequenced in paired-end (2 × 150 bp) mode on an Illumina HiSeq X sequencer. The sequence reads were mapped to rainbow trout reference genome GCF_002163495.1 (Pearse et al., 2019) using BWA-MEM algorithm (Li, 2013), and alignments were converted to BAM (Binary sequence Alignment/Map) format using SAMtools v1.11 (Li et al., 2009). PCR duplicates were marked and removed using Picard v2.18.2 (http://broadinstitute.github.io/picard/). Following our previously published SNP calling and filtering pipeline (Gao et al., 2018), SNPs were called using program freebayes v1.3.1 (Garrison and Marth, 2012), and were annotated using program SnpEff v4.3 (Cingolani et al., 2012).

### Identification of SNPs in the QTL Regions

For the sequence data of parents for the 2015 and 2017 generations, we used a sliding window approach to identify SNPs in the BCWD QTL regions. We used a window size 10,000 bp and a step size 5,000 bp to calculate the fixation index (Fst) value for each window. For individual sequencing, program VCFtools v0.1.16 (Danecek et al., 2011) was used to calculate Fst value for each sliding window. For pooled sequencing, program PoPoolation2 (Kofler et al., 2011) was used to calculate Fst value for each sliding window. Only windows with at least 15 SNPs were included to identify windows with significantly different allele frequencies (empirical *p* < 0.0001) between the R and S groups. For individual sequencing, program VCFtools was also used to calculate Fst value for each SNP with MAF (minor allele frequency) greater than 0.05.

To identify SNPs with significantly different allele frequencies between the pools of R and S offspring from the 2015 generation, Fisher’s exact tests were performed using the program PoPoolation2 with the following settings: -min-coverage 40, --max-coverage 400 and--min-count 10. To correct for multiple tests, SNPs with *p*-values less than 4.05 × 10^–9^ (Bonferroni-correction for 12,338,978 SNPs) were considered as significant.

### SNP Genotyping

Among the SNPs identified by WGS, we selected a set of SNPs in the Omy08 and Omy25 QTL regions for association analyses. The SNPs were selected for assay design because they met one or more of the following criteria: 1) The Fst values between R and S parents were high; 2) The *p*-values of Fisher’s test for different allele frequencies between R and S pools of the 2015 generation were low; 3) The SNPs had high or moderate effects based on SNP annotation; 4) SNPs are near the six SNPs used in our previous haplotype analysis (Liu et al., 2018). The sequences of the selected SNPs were submitted to Fluidigm (South San Francisco, CA) for assay design. After a preliminary evaluation of assay quality using a subset of mapping samples of the 2015 generation, we assembled a panel of 96 SNPs (Supplementary Table S3) to genotype the mapping samples of three consecutive generations, 2015, 2017, and 2019.

We followed the SNP genotyping protocol described in our previous study (Liu et al., 2016). Briefly, DNA samples were pre-amplified, and the pre-amplified products were diluted and used for genotyping with 96.96 Dynamic Array IFCs (Integrated Fluidic Circuits). The arrays were read using EP1 system, and genotypes were called automatically using Fluidigm SNP genotyping analysis software 4.1 with a confidence threshold of 85. The genotype clusters were examined by eye for each assay, and any wrong calls or no calls were corrected manually. The computer program PedCheck (O'Connell and Weeks, 1998) was used to identify genotypes with Mendelian inheritance errors between parents and offspring. Seven SNPs (Supplementary Table S3) were removed from association analysis due to poor genotype clusters or high rates of genotype discrepancies between parents and offspring.

### Family-Based Association Mapping of BCWD Resistance

The program PLINK 1.9 (Chang et al., 2015) was used for family-based association mapping to validate SNPs associated with BCWD resistance (*p* < 0.01). The procedure QFAM was used to analyze the phenotypic data DAYS, and the PERM option was used to correct the family structure. The procedure TDT (transmission disequilibrium test) was used to analyze the binary phenotype STATUS. The association analyses were performed for each of the three consecutive generations, 2015, 2017, and 2019.

### Haplotype Association Analysis of BCWD Resistance

We used three criteria to select three SNPs per QTL region for haplotype association analysis. 1) The selected SNPs are highly associated with BCWD resistance based on single SNP association analysis; 2) The MAF for each selected SNP is greater than 0.2; and 3) The three SNPs for each QTL region span a genomic region less than 0.5 Mb according to rainbow trout reference genome GCF_002163495.1 (Pearse et al., 2019). Based these three criteria, three SNPs, P489, P194, and P355, were selected for the Omy08 QTL, and three SNPs, P420, P430, and P212, were selected for the Omy25 QTL. The same families used for single SNP analysis described above were also used for haplotype association analysis. The haplotypes for each fish were constructed using Beagle 5.1 (Browning and Browning, 2007), and haplotypes with frequency larger than 0.1 were retained for haplotype association analysis. Program PLINK1.9 was used for haplotype association analysis following the same method for family-based association analysis of single SNP as described above.

### Evaluation of MAS for BCWD Resistance in the 2021 Generation

The parents for the 2021 generation were genotyped with 96 SNPs (Supplementary Table S3), and haplotypes for the Omy08 and Omy25 QTL regions were constructed using the same SNPs and method described above. The 163 families of the 2021 generation were sorted by the total count of favorable haplotypes from high to low. The top 25 families were assigned to the high haplotype group, and the bottom 25 families were assigned to low haplotype group. We pooled 10 fish per family by haplotype groups, and the 250 fish were challenged with BCWD in a 40 L tank with a water flow rate of 2.5 L per minute. There were two replicate tanks for each haplotype group. So, a total of 500 fish were challenged per haplotype group. To test the BCWD survival difference between the high and low favorable haplotype groups, a log-rank test was performed using the survival package (Therneau, 2021) available in R version 4.1.2 (R Core Team, 2021).

## Results

### Identification of Genomic Regions Associated With BCWD Resistance Using WGS

**FIGURE 1:**
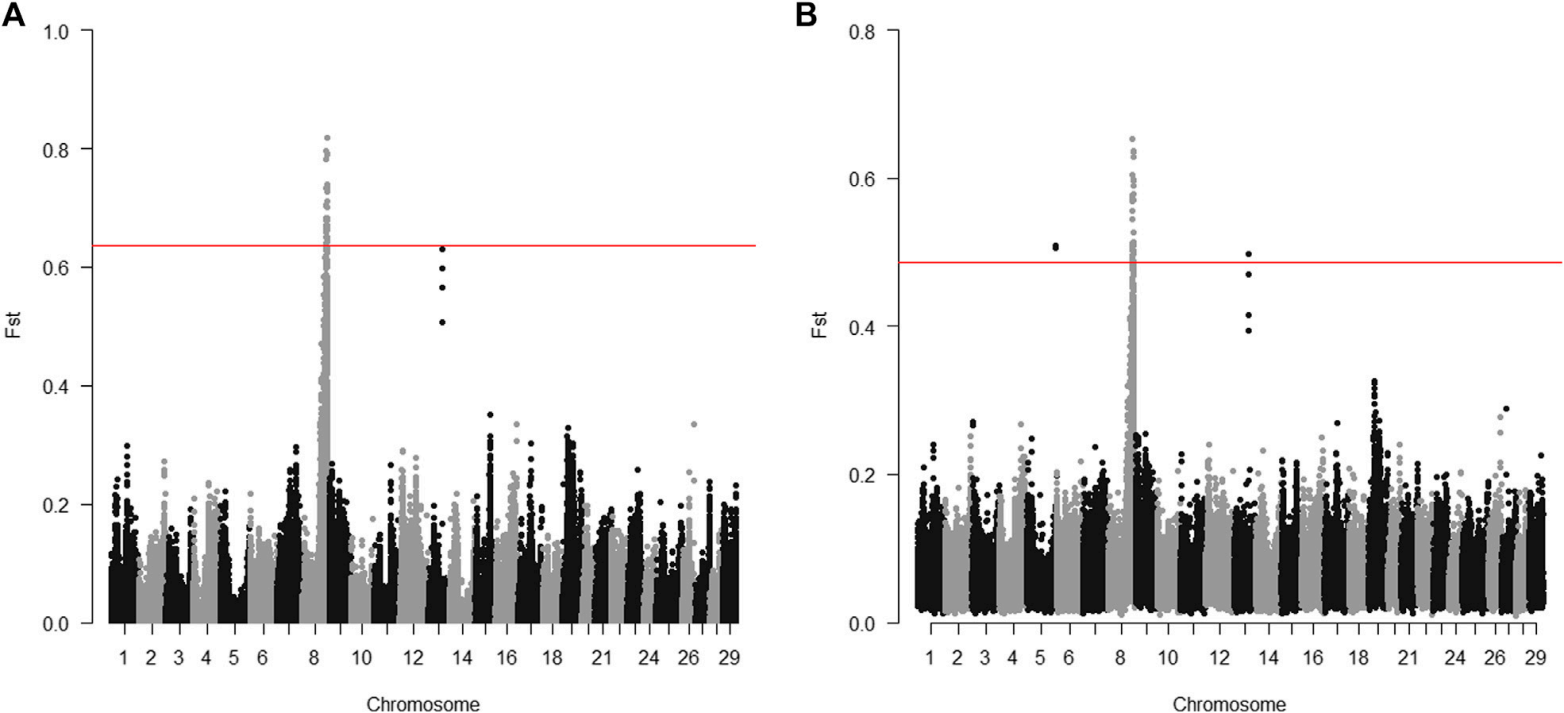
Distribution of Fst between R and S groups of parents for the 2015 generation of the Troutlodge May spawning population. **(A)** sequencing of individuals; **(B)** sequencing of pooled samples. The red horizontal lines represent the threshold for significantly different Fst (empirical *p* < 0.0001).

The 20 parents for the 2015 generation used for sequencing were selected to target the Omy08 QTL for BCWD resistance. For individual sequencing, all windows with significantly different Fst between R and S parents were in a region between 73.2 and 78.2 Mb on chromosome Omy08 (Figure 1A). For pooled sequencing, except two windows on chromosome Omy05 and one window on chromosome Omy13, all the other windows with significantly different Fst between R and S pools were in a region between 73.2 and 78.0 Mb on chromosome Omy08 (Figure 1B). Thus, we selected SNPs in the region from 73.2 to 78.2 Mb to validate SNPs associated with the BCWD QTL on chromosome Omy08.

**FIGURE 2:**
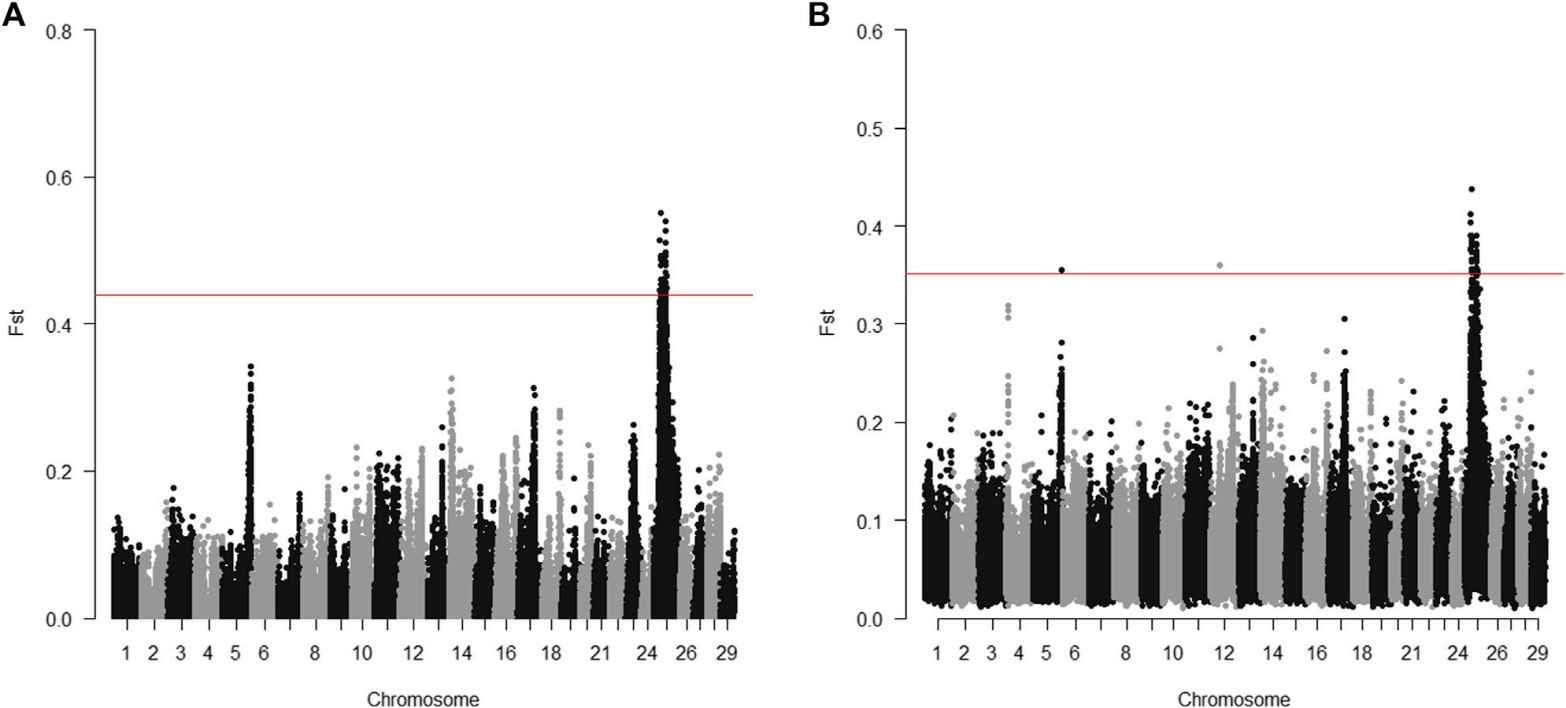
Distribution of Fst between R and S groups of parents for the 2017 generation of the Troutlodge May spawning population. **(A)** sequencing of individuals; **(B)** sequencing of pooled samples. The red horizontal lines represent the threshold for significantly different Fst (empirical *p* < 0.0001).

The 20 parents for the 2017 generation used for sequencing were selected to target the Omy25 QTL for BCWD resistance. For individual sequencing, all windows with significantly different Fst between R and S parents were in a region between positions 16.5 and 40.1 Mb on chromosome Omy25 (Figure 2A). For pooled sequencing, except one window on chromosome Omy05 and one window on chromosome Omy12, all the other windows with significantly different Fst between R and S pools were located on chromosome Omy25 in a region between 18.9 and 41.0 Mb (Figure 2B). Thus, we selected SNPs in the region from 16.5 to 41.0 Mb to validate SNPs associated with the BCWD QTL on chromosome Omy25.

**FIGURE 3:**
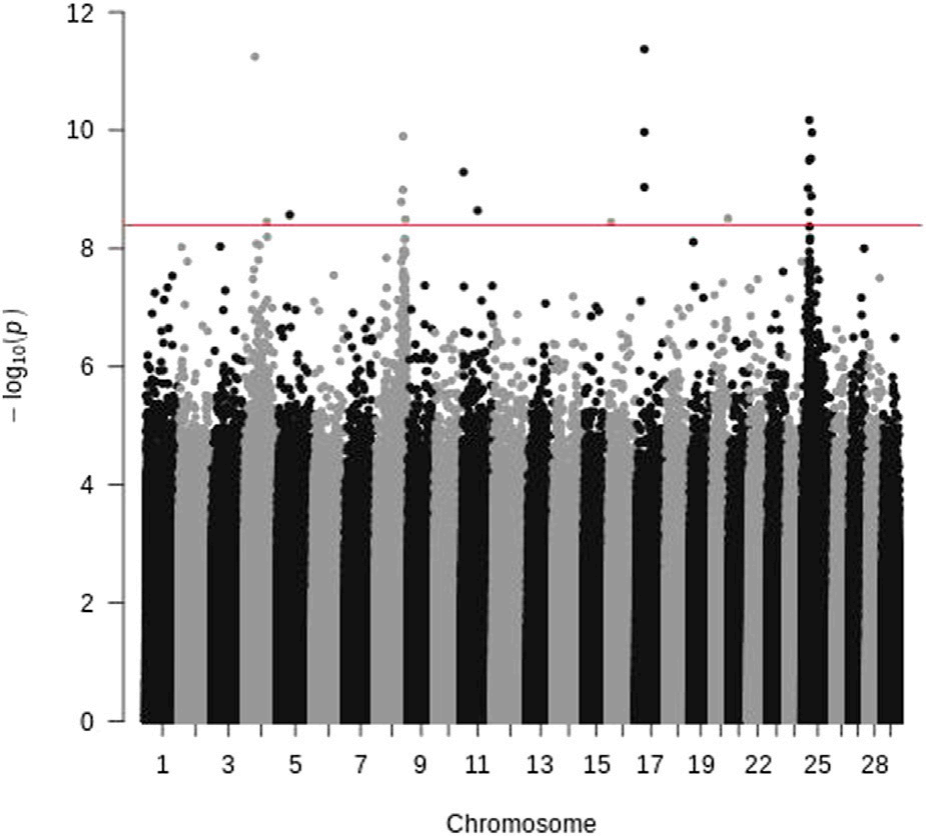
Manhattan plot of pooled offspring of the 2015 generation of the Troutlodge May spawning population. The red horizontal line represents the significance threshold (Bonferroni-correction for 12,338,978 SNPs).

To avoid the potential bias of samples used for sequencing just described above, we also sequenced pooled offspring of the 2015 generation. The samples were selected with BCWD phenotypes alone, and they were pooled by survival status. After mapping the sequence reads to the reference genome, 12.3 million SNPs were identified. Based on Fisher’s test for each SNP, 21 SNPs with significantly different allele frequencies between the R and S pools were identified (Figure 3), and they were located on chromosomes Omy04, Omy05, Omy08, Omy11, Omy16, Omy17, Omy20, and Omy25. Only chromosomes Omy08 and Omy25 had more than three significant SNPs, and these significant SNPs were located in similar QTL regions to those identified by WGS of parents as described above.

### Validation of SNPs Associated With BCWD Resistance

Among the 89 SNPs used for association analysis in three consecutive generations, 2015, 2017, and 2019, 85 SNPs were associated with BCWD resistance in at least one of the three generations (Table 2). Also, 77 out of the 85 validated SNPs were identified via WGS reported in this study. Among the 4 SNPs, P161, P176, P316, and P490, that were not associated with BCWD resistance in this study, P490 was the only SNP derived from WGS.

**TABLE 2:** Validation of SNPs associated with BCWD resistance in three consecutive generations of the Troutlodge May spawning population.

Generation						2015				2017				2019			
Chr.	SNP^a^	Source	Position^b^	Allele1	Allele2	Effect^c^	*p* (DAYS)	OR^d^	*p* (STATUS)	Effect	*p* (DAYS)	OR	*p* (STATUS)	Effect	P (DAYS)	OR	P (STATUS)
3	P178	Liu et al. (2018)	57059195	G	A	2.8	3.7E-04	0.6	1.1E-04	1.3	NS^e^	0.7	7.1E-03	1.9	NS	0.8	NS
8	P318	WGS	75,054,110	T	G	3.6	7.9E-05	0.7	1.7E-03	1.9	2.1E-03	0.7	2.7E-03	−0.3	NS	1.0	NS
8	P319	WGS	75,056,552	G	C	3.5	3.2E-05	0.7	3.3E-03	1.9	4.9E-03	0.7	6.1E-03	−0.9	NS	1.0	NS
8	P323	WGS	75,064,254	A	T	2.8	1.6E-03	0.7	NS	1.9	2.3E-03	0.7	8.5E-03	−0.3	NS	1.0	NS
8	**P489**	WGS	76,457,672	A	C	−4.0	1.0E-04	1.4	NS	-2.9	6.3E-05	1.4	NS	−2.2	NS	1.3	4.5E-03
8	P329	WGS	76,691,975	T	A	−4.0	3.0E-06	1.5	2.7E-04	-2.3	1.7E-03	1.3	NS	−2.5	NS	1.6	1.6E-05
8	P330	WGS	76,692,073	T	A	−4.0	3.0E-06	1.5	2.2E-04	-2.2	2.5E-03	1.3	NS	−2.6	NS	1.6	1.1E-05
8	P332	WGS	76,696,519	C	A	−3.9	2.0E-06	1.4	4.8E-03	-2.9	2.4E-05	1.4	NS	−2.1	NS	1.2	NS
8	**P194**	Liu et al. (2018)	76,747,151	A	G	−4.0	2.0E-06	1.6	3.5E-05	-2.3	2.0E-03	1.3	NS	−2.2	NS	1.5	1.3E-04
8	P341	WGS	76,758,777	A	G	−4.0	2.0E-06	1.5	4.8E-04	−2.6	2.4E-04	1.4	5.0E-03	−1.2	NS	1.2	NS
8	P342	WGS	76,765,345	A	T	−3.9	3.0E-06	1.5	5.8E-04	−2.5	6.4E-04	1.4	7.3E-03	−2.5	8.6E-03	1.3	5.0E-03
8	P346	WGS	76,815,705	G	T	−3.9	3.0E-06	1.5	5.8E-04	−2.1	8.1E-04	0.9	NS	−2.5	8.6E-03	1.5	4.1E-05
8	P347	WGS	76,844,443	G	A	−3.8	4.2E-05	1.4	6.8E-03	−2.4	1.2E-03	1.3	NS	−1.8	NS	1.3	NS
8	P350	WGS	76,853,682	G	T	−4.1	1.2E-05	1.6	3.2E-04	−2.1	5.9E-03	1.2	NS	−1.6	NS	1.6	3.0E-05
8	P354	WGS	76,874,914	C	T	−3.8	4.2E-05	1.4	6.8E-03	−2.4	1.3E-03	1.3	NS	−2.2	NS	1.5	5.2E-05
8	**P355**	WGS	76,875,468	A	G	−3.8	4.2E-05	1.4	6.8E-03	−2.3	1.3E-03	1.5	4.7E-04	−2.2	NS	1.5	5.2E-05
8	P356	WGS	76,879,188	A	G	−3.8	4.2E-05	1.4	6.8E-03	−2.4	1.3E-03	1.3	NS	−2.2	NS	1.5	5.2E-05
8	P357	WGS	76,879,806	C	A	−3.8	4.2E-05	1.4	6.8E-03	−2.4	1.3E-03	1.3	NS	−2.2	NS	1.5	5.4E-05
8	P191	Liu et al. (2018)	78,064,599	A	G	−3.1	3.2E-04	1.2	NS	−2.8	2.3E-03	1.3	NS	−1.2	NS	1.3	NS
13	P494	WGS	42,160,393	C	A	-4.1	2.0E-04	1.4	7.5E-03	−2.5	6.4E-04	1.4	7.3E-03	−2.4	NS	1.4	7.1E-03
25	P381	WGS	17,246,875	C	A	−6.2	2.0E-06	1.6	NS	−2.5	9.1E-03	1.6	2.4E-03	−3.0	8.6E-03	1.5	4.2E-05
25	P382	WGS	17,272,388	A	G	−5.7	3.0E-06	1.5	NS	−2.6	1.7E-03	1.6	7.5E-04	−2.8	8.6E-03	1.5	2.4E-05
25	P383	WGS	17,893,051	T	C	2.5	NS	0.7	NS	−0.8	NS	1.2	NS	1.4	NS	0.8	8.3E-03
25	P384	WGS	17,944,754	T	A	3.7	4.2E-05	0.6	5.2E-03	2.5	5.4E-03	0.6	7.2E-03	4.5	NS	0.3	1.2E-06
25	P386	WGS	18,159,829	A	G	3.7	4.2E-05	0.6	4.1E-03	2.1	9.5E-03	0.7	NS	4.5	NS	0.3	1.2E-06
25	P230	Liu et al. (2018)	18,192,885	G	T	2.5	NS	0.7	NS	2.6	5.1E-04	0.6	2.1E-04	2.7	NS	0.6	2.7E-06
25	P391	WGS	19,063,776	A	G	−5.5	3.0E-06	2.1	8.0E-06	−3.0	1.4E-04	1.7	3.1E-04	−3.0	NS	1.6	1.7E-06
25	P392	WGS	19,131,282	A	G	−6.3	1.0E-06	2.0	5.4E-08	−3.7	3.0E-06	1.7	3.5E-05	−2.6	NS	1.5	1.3E-06
25	P393	WGS	19,285,578	T	C	−6.7	1.0E-06	1.9	1.7E-05	−3.4	1.5E-04	1.6	1.2E-03	−2.6	NS	1.4	2.1E-04
25	P395	WGS	19,356,106	A	T	−5.5	1.0E-06	1.9	2.8E-05	−2.9	1.9E-04	1.6	3.1E-04	−3.4	2.3E-03	1.7	2.8E-09
25	P398	WGS	19,480,661	A	G	3.9	3.0E-05	0.6	1.4E-03	2.8	1.5E-03	0.6	7.2E-03	4.8	NS	0.3	4.2E-07
25	P399	WGS	19,525,929	G	T	-6.3	1.0E-06	2.1	5.9E-08	−3.8	1.0E-06	1.7	2.0E-05	−2.7	NS	1.5	1.1E-06
25	P214	Liu et al. (2018)	19,553,268	C	A	4.1	1.0E-06	0.7	1.1E-03	2.8	2.0E-04	0.7	6.9E-03	3.2	3.8E-03	0.6	1.4E-08
25	P402	WGS	19,652,654	T	A	-6.1	1.0E-06	2.0	2.1E-07	-3.6	1.0E-06	1.7	2.6E-05	-3.3	2.3E-03	1.7	4.0E-09
25	P404	WGS	19,682,358	G	A	4.1	1.0E-06	0.7	1.1E-03	2.9	8.1E-05	0.7	5.0E-03	3.5	7.2E-03	0.6	4.8E-09
25	P409	WGS	19,886,570	C	T	3.9	1.4E-05	0.6	2.0E-04	1.2	NS	0.8	NS	1.5	NS	0.7	NS
25	P412	WGS	20059482	C	A	−5.9	1.0E-06	1.9	1.1E-06	−3.6	4.0E-06	1.7	1.2E-04	−2.6	NS	1.5	5.1E-07
25	P413	WGS	20,082,809	T	C	−5.5	1.0E-06	1.9	5.7E-05	−3.0	1.9E-04	1.6	3.1E-04	−3.6	4.3E-03	1.8	5.9E-09
25	P415	WGS	20,153,489	C	T	4.1	1.5E-05	0.6	1.6E-03	1.6	NS	0.7	7.2E-03	3.3	NS	0.5	2.1E-05
25	P417	WGS	20,228,457	C	T	3.5	7.0E-06	0.7	4.1E-03	1.8	NS	0.9	NS	2.4	NS	0.7	8.6E-05
25	**P420**	WGS	20,306,455	G	A	4.0	1.0E-06	0.7	5.8E-03	3.2	2.1E-05	0.8	NS	3.6	7.2E-03	0.5	1.0E-09
25	P495	WGS	20,307,827	C	A	−6.0	1.1E-05	1.9	4.4E-05	−3.7	1.0E-06	1.7	1.2E-04	−2.5	NS	1.5	2.2E-06
25	P424	WGS	20,403,696	C	T	−5.9	1.0E-06	1.9	1.1E-06	−3.6	1.0E-06	1.7	1.2E-04	−2.5	NS	1.5	4.9E-07
25	P425	WGS	20,437,940	C	T	−6.2	2.9E-05	1.6	NS	−2.8	3.0E-03	1.6	4.6E-03	−3.2	NS	1.6	8.9E-05
25	P426	WGS	20,465,120	G	A	−5.7	2.9E-05	1.6	NS	−2.6	NS	1.4	NS	−2.5	NS	0.9	NS
25	P429	WGS	20,570,095	C	T	−6.6	8.5E-06	1.8	4.3E-03	−3.4	NS	1.3	NS	0.0	NS	1.1	NS
25	**P430**	WGS	20,591,515	T	G	−5.1	9.4E-05	1.9	1.6E-04	−2.9	2.3E-04	1.6	1.4E-03	−3.3	NS	1.8	1.2E-07
25	P431	WGS	20,647,628	A	G	−5.1	9.4E-05	1.9	1.6E-04	−2.9	2.3E-04	1.6	1.4E-03	−3.4	NS	1.8	4.6E-08
25	P433	WGS	20,722,720	A	G	−5.9	1.0E-06	1.9	1.1E-06	−3.6	1.0E-06	1.7	1.2E-04	−2.6	NS	1.5	1.5E-06
25	**P212**	Liu et al. (2018)	20,751,780	T	G	−5.9	1.0E-06	1.9	1.6E-06	−3.6	1.0E-06	1.6	1.5E-04	−2.6	NS	1.5	1.5E-06
25	P435	WGS	20,770,014	T	G	−6.0	1.0E-06	1.9	1.1E-06	−3.7	1.0E-06	1.7	1.2E-04	−2.6	NS	1.5	1.4E-06
25	P436	WGS	20,797,381	C	A	−4.0	1.3E-04	1.6	1.1E-04	−3.1	7.0E-06	1.4	9.2E-04	−2.1	NS	1.4	2.2E-05
25	P437	WGS	20,814,089	G	A	−5.6	1.0E-06	1.8	1.3E-04	−3.1	8.1E-05	1.7	1.8E-04	−3.5	4.3E-03	1.7	3.3E-09
25	P438	WGS	20,905,605	T	C	−6.1	1.0E-06	1.8	3.1E-06	−3.8	1.0E-06	1.8	3.2E-07	−3.2	NS	1.7	1.5E-09
25	P439	WGS	20,918,179	C	T	−5.1	9.4E-05	1.9	1.6E-04	−2.9	2.0E-04	1.6	1.4E-03	−3.2	NS	1.7	4.4E-08
25	P440	WGS	20,937,844	A	T	3.9	3.0E-05	0.6	2.4E-03	2.5	5.9E-03	0.7	NS	4.8	NS	0.3	4.2E-07
25	P443	WGS	21,034,905	T	G	4.2	1.2E-05	0.6	2.1E-03	1.4	NS	0.7	7.2E-03	4.9	NS	0.3	1.4E-07
25	P446	WGS	21,118,315	A	C	−5.5	3.0E-06	1.8	1.3E-04	−2.7	5.6E-04	1.5	6.0E-03	−3.5	4.3E-03	1.7	1.2E-08
25	P228	Liu et al. (2018)	21,146,360	T	G	−5.6	2.0E-06	1.9	8.7E-05	−3.0	8.7E-05	1.7	1.8E-04	−3.4	2.3E-03	1.7	2.1E-09
25	P447	WGS	21,164,811	A	G	−6.2	2.9E-05	1.6	NS	−2.8	4.1E-03	1.6	5.8E-03	−3.2	NS	1.6	1.2E-04
25	P497	WGS	21,245,059	T	C	3.0	2.9E-03	0.7	NS	2.7	3.1E-03	0.7	NS	4.9	NS	0.3	1.4E-07
25	P448	WGS	21,245,244	T	C	3.8	7.4E-05	0.6	2.4E-03	2.7	3.1E-03	0.7	NS	2.5	NS	0.5	6.0E-04
25	P498	WGS	21,260,524	T	G	3.6	3.5E-04	0.8	NS	2.9	2.0E-05	0.7	1.3E-04	3.4	4.0E-03	0.6	9.4E-09
25	P499	WGS	21,260,707	C	T	3.0	3.2E-03	0.7	NS	2.6	5.0E-03	0.7	NS	4.9	NS	0.3	9.0E-08
25	P451	WGS	21,429,395	T	C	−6.4	6.5E-06	1.7	4.8E-03	−3.7	3.9E-03	1.5	NS	−3.8	NS	1.7	2.0E-03
25	P360	WGS	21,430,497	G	T	3.4	1.5E-05	0.7	6.2E-03	1.5	NS	0.9	NS	2.7	NS	0.7	1.0E-05
25	P361	WGS	21,431,434	C	T	3.7	2.0E-06	0.7	9.3E-03	3.0	1.3E-04	0.8	NS	3.0	NS	0.6	2.9E-08
25	P363	WGS	21,468,711	C	G	3.8	2.0E-06	0.7	NS	2.7	3.7E-04	0.9	NS	2.9	NS	0.6	1.8E-08
25	P372	WGS	21,469,214	T	G	3.9	2.0E-06	0.7	8.3E-03	3.4	1.4E-05	0.7	NS	3.5	NS	0.6	2.7E-10
25	P222	Liu et al. (2018)	21,530,601	A	C	2.5	NS	0.5	NS	2.8	3.4E-03	0.7	NS	4.9	NS	0.3	9.0E-08
25	P453	WGS	21,535,437	A	G	−6.2	2.9E-05	1.6	NS	−2.9	2.9E-03	1.6	4.6E-03	−3.2	NS	1.6	6.0E-05
25	P458	WGS	21,955,099	T	A	3.7	1.3E-04	0.6	2.4E-03	2.5	2.2E-03	0.7	NS	4.9	NS	0.3	4.2E-07
25	P374	WGS	22,346,077	G	T	4.0	2.0E-06	0.7	6.0E-03	3.3	9.0E-06	0.8	NS	3.6	7.2E-03	0.6	4.4E-09
25	P369	WGS	22,363,310	T	G	3.6	3.0E-06	0.7	9.5E-03	3.3	1.9E-05	0.8	NS	3.2	NS	0.6	4.4E-08
25	P371	WGS	22,373,578	A	C	3.6	6.0E-06	0.7	6.1E-03	2.2	4.0E-03	0.8	NS	3.3	NS	0.6	5.9E-07
25	P468	WGS	22,900,574	A	C	3.3	2.7E-05	0.7	NS	2.3	1.5E-03	0.9	NS	3.5	7.2E-03	0.6	1.1E-07
25	P469	WGS	22,929,112	G	A	−2.8	8.7E-04	1.4	4.1E-03	−1.7	NS	1.2	NS	-3.6	NS	1.6	1.9E-05
25	P470	WGS	22,954,748	T	G	−5.4	2.1E-05	1.5	NS	−3.4	6.3E-05	1.7	1.4E-04	-3.4	4.0E-03	1.6	3.4E-07
25	P472	WGS	23,514,396	T	C	3.4	5.6E-05	0.6	1.8E-04	0.8	NS	0.8	NS	-1.0	NS	1.1	NS
25	P473	WGS	23,533,980	T	C	4.3	1.0E-06	0.6	1.1E-05	2.3	1.8E-03	0.6	6.1E-04	2.9	NS	0.5	1.7E-05
25	P476	WGS	23,741,596	C	T	3.8	6.2E-05	0.6	2.4E-03	2.6	1.1E-03	0.7	NS	4.5	NS	0.3	2.4E-06
25	P481	WGS	24,385,764	A	G	3.9	2.9E-05	0.6	2.4E-03	2.7	1.1E-03	0.7	NS	4.9	NS	0.3	1.8E-06
25	P482	WGS	24,459,019	A	C	4.4	1.0E-06	0.6	1.1E-05	2.3	3.0E-03	0.7	1.2E-03	2.9	NS	0.5	3.3E-05
25	P483	WGS	25,069,717	A	T	−4.9	6.7E-05	1.8	9.4E-04	−2.0	NS	1.3	NS	-2.3	NS	1.5	NS
25	P485	WGS	25,580,318	T	G	3.9	1.3E-05	0.6	1.6E-03	2.0	NS	0.8	NS	2.5	NS	0.6	8.8E-03

a SNPs, used for haplotype analysis (Table 3) are highlighted in bold.

b SNP, position on rainbow trout reference genome GCF_002163495.1.

c Allele substitution effect, positive number indicates that allele 1 increases the number of survival days, and negative number indicates that allele 1 reduces the number of survival days.

d Odds ratio, greater than one indicates that allele 1 increases the risk of death from BCWD, and less than one indicates that allele 1 reduces the risk of death from BCWD.

e Not significant (*p* > 0.01).

### Refined Haplotypes Associated With BCWD Resistance

The 85 SNPs associated with BCWD resistance validated above allowed us to reduce the physical size of the haplotypes associated with BCWD resistance. Using the three criteria described in the method section, we selected SNPs, P489, P194, and P355, for the Omy08 QTL, and SNPs, P420, P430, and P212, for the Omy25 QTL. The three selected SNPs span a region less than 0.5 Mb. Thus, we reduced the sizes of haplotypes by about two-thirds. The results of haplotype association analysis are summarized in Table 3. For the Omy08 QTL, haplotype CGG was associated with BCWD resistance in all three generations, which increases the number of survival days and reduces the risk of death from BCWD. Similarly, haplotype GGG on chromosome Omy25 was also associated with BCWD resistance in all three generations, which increases the number of survival days and reduces the risk of death from BCWD. The other two haplotypes (Table 3) were associated with BCWD susceptibility. Because the goal of selective breeding is to improve BCWD resistance, we will focus on the two haplotypes associated with BCWD resistance and refer to them as favorable haplotypes for BCWD resistance.

**TABLE 3:** Haplotype association analysis of BCWD resistance in three consecutive generations of the Troutlodge May spawning population.

Generation		2015				2017				2019			
Chr.	Haplotype	Effect^a^	*p* (DAYS)	OR^b^	*p* (STATUS)	Effect	P (DAYS)	OR	P (STATUS)	Effect	P (DAYS)	OR	P (STATUS)
8	CGG	3.8	4.0E-06	0.6	2.9E-04	2.1	2.9E-03	0.7	1.5E-02	2.8	7.6E-03	0.7	9.9E-04
8	AAA	−3.9	2.7E-05	1.2	NS^c^	−2.8	2.3E-03	1.1	NS	−1.3	NS	1.0	NS
25	GGG	4.0	1.0E-06	0.7	4.1E-03	3.0	4.6E-05	0.8	1.4E-02	3.5	8.8E-03	0.6	3.1E-09
25	ATT	−5.1	7.3E-05	1.9	1.6E-04	−2.9	1.6E-04	1.6	1.9E-03	−3.2	NS	1.7	1.1E-06

a Positive number indicates that the haplotype increases the number of survival days, and negative number indicates that the haplotype reduces the number of survival days.

b Odds ratio, greater than one indicates that the haplotype increases the risk of death from BCWD, and less than one indicates that the haplotype reduces the risk of death from BCWD.

c Not significant (*p* > 0.05).

### Evaluation of MAS for BCWD Resistance

**FIGURE 4:**
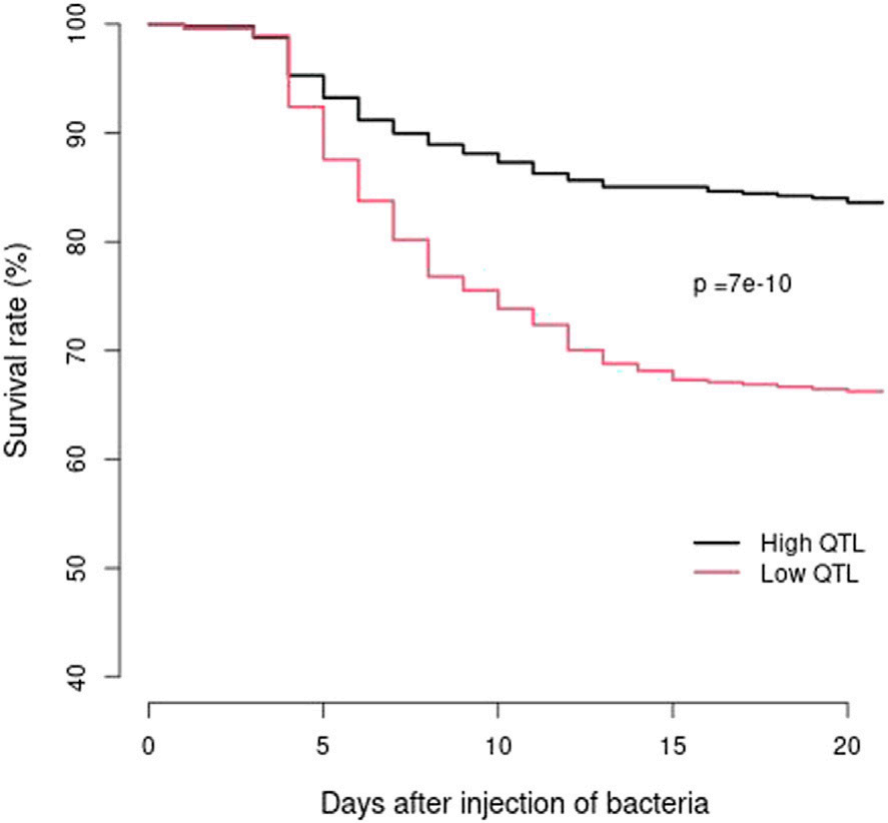
BCWD survival curves of the 2021 generation of the Troutlodge May spawning population. The black curve represents the families with high counts of favorable haplotypes for the two major BCWD QTL regions. The red curve represents the families with low counts of favorable haplotypes for the two major BCWD QTL regions.

The 25 families from the 2021 generation with high or low counts of favorable haplotypes for the two major QTL regions had an average of 6.48 and 2.56 favorable haplotypes per family, respectively. The BCWD survival rates for the pooled families with high or low counts of favorable haplotypes were 83.6% and 66.2%, respectively. Survival analysis demonstrated that the two BCWD survival curves were significantly different (p = 7e-10) (Figure 4) for the two groups of families with high or low counts of favorable haplotypes. Thus, the favorable haplotypes can be used to select families with improved BCWD resistance.

## Discussion

In this study, we used WGS to identify additional SNPs associated with the two major QTL for BCWD resistance, and 77 SNPs identified from WGS were validated by association mapping in three consecutive generations of the Troutlodge May spawning population. The additional SNPs associated with BCWD resistance allowed us to refine the favorable haplotypes associated with BCWD resistance. We demonstrated that the refined favorable QTL haplotypes can be used for MAS for BCWD resistance in the Troutlodge May spawning population.

### Identification of SNPs Associated With BCWD Resistance Using WGS

Among the 78 SNPs derived from WGS, only SNP P490 was not associated with BCWD resistance in this study. The high SNP validation rate was largely due to several factors. 1) The samples used for sequencing were selected with two methods, BCWD phenotypes together with QTL haplotypes or BCWD phenotypes alone; 2) We used two sequencing strategies, sequencing of individuals and pooled samples; 3) The SNPs selected for assay design were filtered by multiple criteria as described in the method section; 4) We removed SNPs with poor genotype quality or SNPs were not associated with BCWD resistance based on a preliminary study with a sub-set of mapping samples. Due to the genotyping platform used in this study, only 96 SNPs including both SNPs derived from WGS and SNPs reported in our previous study (Liu et al., 2018) were used for association mapping. However, analysis of WGS revealed many more SNPs that were putatively associated with BCWD resistance. Thus, WGS is a powerful tool to identify SNPs associated with BCWD resistance in rainbow trout.

Sequencing pools of individuals (pool-seq) is cost-effective, and has been successfully applied to a variety of studies (Schlotterer et al., 2014). However, pool-seq also has technical challenges and limitations. Unequal representation of DNA samples in the pools can cause false positive signals. The same parental DNA samples were sequenced individually and by pool-seq in this study. Although the genomic regions with significantly different Fst were similar for both sequencing strategies (Figures 1, 2) for the targeted QTL regions, a few additional genomic regions also showed significantly different allele frequencies for the pooled samples, which was likely due to unequal representation of DNA samples in the pools. Compared to the results of sequencing of parents, more genomic regions showed significantly different allele frequencies between the DNA pools of offspring in the 2015 generation (Figure 3). Most of them are likely false positives due to the technical challenges of pool-seq. In addition to the possibility of unequal representation of each offspring in the pool, unreliable BCWD phenotypes could be a major factor since it is not possible to have replicated BCWD challenges of an individual fish. The offspring were selected for pool-seq on basis of BCWD survival status of individual fish. On the other hand, the family BCWD survival rates are based on a large number of offspring, and hence are much more reliable than the BCWD survival status of an individual fish.

### Two Robust QTL for BCWD Resistance in Rainbow Trout

QTL validation is essential for implement of MAS in breeding programs and identification of causative genes. The two major QTL for BCWD resistance, located on chromosomes Omy08 and Omy25, were initially identified in the 2013 generation of Troutlodge May spawning population (Vallejo et al., 2017b), and were validated in the 2015 generation of the same population (Liu et al., 2018). In this study, we used WGS to identify additional SNPs associated with these two major QTL, and 77 additional SNPs associated with BCWD resistance were validated in three consecutive generations, 2015, 2017 and 2019, of the Troutlodge May spawning population. Thus, the two major QTL for BCWD resistance are robust in the Troutlodge May population, and it is worthwhile to evaluate MAS for BCWD resistance and to identify positional candidate genes underlying the QTL.

### Robust MAS for BCWD Resistance in Rainbow Trout

We reported previously that the accuracies of retrospective MAS for BCWD resistance using favorable haplotypes associated with the two major BCWD QTL were equal or greater than the accuracies of family-based selection in the same generation of odd-year Troutlodge May spawning population (Liu et al., 2018). In this study, we reduced the physical size of the haplotypes by about two-thirds. We then used the refined haplotypes for MAS for BCWD resistance in the 2021 generation of the Troutlodge May spawning population. Based on the QTL haplotypes of the parents, two groups of families with high or low counts of favorable haplotypes, respectively, were selected for pooled BCWD challenge. The two groups of families had significantly different BCWD survival curves. It is notable that the odd-year and even-year Troutlodge May spawning populations have been combined into one population since the 2019 generation. Together with the results of retrospective MAS reported previously (Liu et al., 2018), we conclude that MAS for BCWD resistance is robust in the Troutlodge May spawning population.

Although we focused on MAS for BCWD in this study, it is important to note that the additional SNPs associated BCWD resistance reported in this study should also be useful to improve the accuracy of GS for BCWD resistance. We reported previously that the accuracy of GS for BCWD resistance using 70 SNPs associated with BCWD resistance was similar to the accuracy of the whole-genome 57K SNP array (Vallejo et al., 2018). Furthermore, it has been documented that functional and causative variants can be used to improve the accuracy of GS (Xiang et al., 2021). Some of the SNPs reported in this study are located within candidate genes for BCWD resistance (see below).

### Candidate Genes of QTL for BCWD Resistance in Rainbow Trout

Our long-term goal is to identify causative genes for BCWD resistance in rainbow trout. Although the refined haplotypes are associated with resistance to BCWD, they may or may not span the QTL regions. Thus, we arbitrarily extended 0.5 Mb on each end of the refined favorable haplotypes associated with BCWD resistance, and then examined protein-coding genes in the corresponding regions of rainbow trout Arlee reference genome (Gao et al., 2021), which has a better genome coverage than the previous Swanson reference genome (Pearse et al., 2019). Based on the NCBI rainbow trout gene annotation release 101, a total of 70 annotated protein-coding genes were identified in the two major QTL regions (Supplementary Table S4).

Among the 40 annotated protein-coding genes in the Omy08 QTL region, multiple genes are likely related to immune responses. Both LOC110530755 and LOC110530756 encode NACHT proteins, which are implicated in apoptosis and MHC (major histocompatibility complex) transcription activation (Koonin and Aravind, 2000; Laing et al., 2008), and play important roles in activation of animal innate immune responses to pathogen infection (Jones et al., 2016). Furthermore, NACHT proteins such as Nod like receptors also play an important role in activation of pyroptosis pathway in both mammals and fish (Song et al., 2022). Three other candidate genes, LOC110530758, LOC110530759, and LOC110530764, encode proteins likely belonging to the signaling lymphocytic activation molecule (SLAM) family of receptors, which in mammals are critical elements for both innate and adaptive immune responses (Veillette, 2006; Cannons et al., 2011). Also, these three SLAM genes were modestly upregulated at day 5 post challenge with *Flavobacterium psychrophilum* in the study reported by Marancik et al. (2015). In addition to the putative functions, the results of association mapping (Table 2) also indicated that these genes are strong candidates for the Omy08 QTL. All 8 SNPs in the candidate gene regions (Supplementary Table S4) are significantly associated with BCWD resistance in three consecutive generations of the Troutlodge May spawning population (Table 2). Thus, these immune-related genes are good candidates for the Omy08 QTL for BCWD resistance.

Among the 30 annotated genes in the Omy25 QTL region, gene LOC100136157, which encodes invariant chain INVX, stands out as a promising candidate gene for this QTL. For simplicity and consistency with rainbow trout literatures, we refer this gene as INVX from now on. Rainbow trout INVX was initially cloned and characterized by Fujiki et al. (2003), and is a homolog of mammalian invariant chain genes, which play important roles in antigen presentation (Schroder, 2016). Transcript level of INVX in rainbow trout cell line culture was significantly increased at 96 and 120 h after immune system activation with PMA (phorbol 12-myristate 13-acetate) (Semple et al., 2019). Also, INVX protein level was significantly reduced at 168 h after PMA stimulation (Semple et al., 2019). In addition to the putative function of INVX, there is also another line of evidence supporting INVX as a candidate gene for the Omy25 QTL. SNP P446, located in the intron of gene INVX, was significantly associated with BCWD resistance in three consecutive generations of the Troutlodge May spawning population (Table 2). Therefore, we will continue to evaluate this candidate gene using other approaches in the future.

In addition to the candidate genes highlighted above, we would like to caution that other genes could also be candidate genes for the BCWD QTL. Although we focused on protein-coding genes, there are also two annotated non-coding RNA genes in the Omy08 QTL region. We should not completely rule out other genes in the QTL regions until we can identify with high confidence the causative genes underlying the two QTL for BCWD resistance in rainbow trout.

## Conclusion

WGS is a powerful tool to identify additional SNPs associated with the two major QTL for BCWD resistance in rainbow trout. The additional SNPs allowed us to reduce the physical size of haplotypes associated with BCWD resistance. We also demonstrated that the refined favorable QTL haplotypes can be used for MAS for BCWD resistance in the Troutlodge May spawning population. Thus, the additional SNPs and refined haplotypes associated with BCWD resistance reported in this study are useful for improvement of BCWD resistance and for eventual identification of genes for BCWD resistance in rainbow trout.

## Data Availability

The datasets presented in this study can be found in online repositories. The names of the repository/repositories and accession number(s) can be found in the article/Supplementary Material.

## Supplementary Material

Supplementary Table S1 Detailed information of BCWD challenge experiments of four consecutive generations of the Troutlodge May spawning population.

Supplementary Table S2 The 40 parents of the 2015 and 2017 generations of the Troutlodge May spawning population selected for sequencing.

Supplementary Table S3 The primer sequences of 96 SNPs used for association mapping in three consecutive generations of the Troutlodge May spawning population.

Supplementary Table S4 Protein-coding genes in the two major BCWD QTL regions based on NCBI rainbow trout gene annotation release 101.
